# Predicting Cognitive Decline in Parkinson’s Disease: Can We Ask the Genes?

**DOI:** 10.3389/fneur.2014.00224

**Published:** 2014-10-27

**Authors:** Fabiola De Marchi, Miryam Carecchio, Roberto Cantello, Cristoforo Comi

**Affiliations:** ^1^Department of Neurology, University of Eastern Piedmont, Novara, Italy

**Keywords:** dementia, biomarkers, genetics, single nucleotide polymorphism, disease progression

## Introduction

Parkinson’s disease dementia (PDD) is characterized by progressive cognitive decline, mainly affecting executive functions, which occurs in PD patients at least 1 year after the onset of motor symptoms, when no other causes of dementia can be detected. Such temporal cut-off allows to discriminate between PDD and Lewy body disease (LBD) in which dementia is present since the disease onset, along with parkinsonism, visual hallucinations, and fluctuations in the level of consciousness. The prevalence of dementia in patients with PD is 25%, increasing up to 80% in patients with late onset after 20 years of follow-up. In contrast, PDD has been detected in only 20% of young onset PD patients after a follow-up of more than 18 years (1).

Clinical criteria for probable PDD require a diagnosis of PD (2) and a slowly progressive dementia syndrome. Typical cognitive deficits in two of four domains (attention, executive function, visuospatial function, and free recall) and at least one behavioral symptom (apathy, depression/anxious mood, hallucinations, delusions, or excessive daytime sleepiness) must be present. Exclusion criteria include unknown time interval between motor and cognitive symptoms, acute confusion, resulting from systemic diseases or drug intoxication and features compatible with vascular dementia (3).

In recent years, several studies have focused on predictive markers of cognitive decline in PD. Clinical, neuroimaging, and molecular markers have been identified. Nonetheless, which markers are most reliable and applicable to clinical practice to predict the long-term prognosis of these patients still needs to be clarified.

## Potential Disease Markers

Higher age at onset and gait impairment are the best clinical predictors of PDD. Community-studies showed that gait impairment both precedes and predicts dementia. Moreover, a direct correlation between brain atrophy and freezing of gait has been detected (4).

Neuroimaging studies have demonstrated that PDD is associated with extensive cortical atrophy, which may be quantified with structural MRI. More detailed imaging studies can differentiate PD patients with mild cognitive impairment (PD-MCI) from PDD. Voxel based morphometry (VBM) shows that PD-MCI patients display selective posterior cortical atrophy, while PDD patients have a more widespread cortical atrophy involving the posterior and temporal lobes, hippocampus, and frontal association areas (5). 18FDG PET in PDD patients shows a pattern of hypometabolism targeting parietal, occipital, temporal, frontal lobes, and anterior cingulate cortex, which is directly proportional to disease severity (6). DaTSCAN SPECT shows that loss of mesolimbic and mesocortical dopaminergic function is likely to be more relevant to PDD than DLB (7).

The cerebrospinal fluid (CSF) provides relevant information on the central nervous system (CNS) biochemical environment (8), and its study in neurodegenerative conditions has boosted since the discovery of changes in beta amyloid/tau concentrations in the CSF of Alzheimer’s disease (AD) patients (9). Increased CSF tau protein levels are typical of AD, but they are also observed in other neurodegenerative diseases with rapid neuronal cell loss, even independently from tau-related pathology (10). Therefore, CSF tau should be considered a non-specific marker of neuronal degeneration (11). CSF tau levels do not differ in PD patients and controls, whereas PDD patients display higher levels compared to PD patients but lower than LBD and AD patients. On the contrary, CSF levels of phosphorylated tau are not useful to differentiate these conditions (12). α-synuclein, the major constituent of Lewy bodies, was found to be decreased in the CSF of PD patients at diagnosis compared to controls. Such decrease was particularly evident in patients with akinetic-rigid PD. On the contrary, no correlation between α-synuclein levels and disease progression was found and no specific comparison between PD and PDD patients was performed (13). Aβ-peptides are the major constituents of amyloid plaques. Decreased CSF levels of Aβ1–42 in PDD patients were strongly associated with cognitive decline over time, in particular, with a faster decline in cognitive performances assessed by the dementia rating scale (DRS) (14). Osteopontin (OPN), a pro-inflammatory molecule previously found to be associated with AD progression (15), has also been studied in PD and LBD (16). OPN serum and CSF levels are higher in PD patients than controls, with CSF levels positively correlating with concomitant dementia (17). Accordingly, similar findings were reported in LBD, where a single nucleotide polymorphism (SNP) of the OPN gene was also shown to be associated to disease susceptibility (18).

With regard to studies on serum and plasma, just a few significant associations between levels of circulating molecules and PDD have been detected so far. In a study assessing baseline plasma levels of 102 proteins in PD patients, only decrease of epidermal growth factor (EGF) was found to correlate to poor cognitive scores at baseline and predict a major risk of cognitive decline (19). More recent studies have also suggested association between serum levels of uric acid (20) and plasma homocysteine (21) and the development of dementia, but such results need replication in larger cohorts.

The potential markers for PDD development that we have reviewed so far have, however, some downsides: neuroimaging studies are generally not invasive but they are very expensive and require advanced technology, highly specialized operators and patients’ collaboration for a long period of time.

Studies on body fluids are generally less expensive than imaging, but in case of CSF studies, they require an invasive procedure, with potential risks, especially in the elderly. Furthermore, circulating molecules can serve as prognostic markers only if the sample is obtained at the time of diagnosis, when patients are drug-naive and the disease is in its early phase. In fact, biological molecules may undergo biochemical changes during the evolution of the disease and due to pharmacological treatment (22).

In recent years, proteomic techniques have been increasingly used to explore the molecular mechanisms underlying PD and to detect biomarkers allowing early diagnosis, prediction of disease progression, and a more targeted treatment. Proteomics can be performed on CSF, plasma, or circulating cells (23). Nonetheless, it is generally quite expensive and no definite biomarker has entered the routine clinical practice so far.

## Role of Genetics

Genetic studies may provide ideal markers for predicting disease progression as DNA sequence is not likely to undergo changes during disease course and is not influenced by drug treatment. Preliminary prognostic correlations can be obtained with cross-sectional or even retrospective data. To this respect, the genetic background of PDD has been explored with both unbiased and candidate–gene approaches. Although most genome wide association studies (GWAS) performed so far were focused on PD susceptibility, Chung et al. analyzed the common variants associated with motor and cognitive progression in 443 PD subjects. Two potential markers of progression were identified: SNP rs6482992 of CLRN3 and SNP rs10958605 of C8orf4: the former provided the best prediction of cognitive deterioration [hazard ratio 1.81, *p* = 1.81 × 10(−6)], the latter, which is likely involved in neuroinflammatory pathways (8), was associated with both motor and cognitive outcomes.

Traditional candidate–gene approaches have mainly focused on genes involved in pathways of neurodegeneration, such as mitochondrial transcription factor A (TFAM) or brain derived neurotrophic factor (BDNF); alternatively, genes like apolipoprotein E (APOE) and microtubule-associated protein Tau (MAPT), were selected among those previously associated with AD (24).

A SNP in the gene encoding TFAM, A > G rs2306604, has been recently studied in PDD patients compared to controls. Data showed that the A allele was associated with PDD (*p* = 0.024, OR = 2.092), even though such findings need to be replicated in a larger population (25).

Brain derived neurotrophic factor is a trophic factor that can act as synaptic modulator and interact with dopaminergic transmission and dopamine receptor stimulation in the fronto-striatal circuitry, whose integrity is crucial for the cognitive aspects of PD. A SNP in the BDNF gene, namely, G196A, resulting in a methionine (Met)–valine (Val) substitution at codon 66 (Val66Met), was shown to be associated with increased susceptibility to AD (26). Intriguingly, PD patients carrying AA genotype of G196A display significantly increased risk of cognitive decline (27).

The ε4 allele of APOE was shown to be associated with higher prevalence of dementia in PD patients. A meta-analysis indicated an over-representation of APOE ε4 carriers in PDD patients compared to non-demented cases [OR 1.74 (1.36–2.23)] (28). To further support these findings, two recent reports confirmed that APOE ε4 is an important predictor of cognitive function in PD across multiple domains (i.e., recall and delayed recall, semantic verbal fluency) (29) and it also has a relevant influence on the memory domain (30).

The H1/H1 haplotype of MAPT was associated with an increased risk of several neurodegenerative disorders. A possible association between the H1 allele and cognitive decline in PD was explored, but the results obtained were conflicting, with some studies showing a positive correlation between PDD and the H1 allele, in particular, with the sub-haplotype H1p (31), and others that provided negative results (32).

Important insights into the relationship between genetics and the cognitive progression of PD derive from the natural history of familial cases. PARKIN and leucine-rich repeat kinase 2 (LRRK2) mutations are associated to a lower rate of PDD compared to sporadic cases, whereas opposite findings were shown for α-synuclein (*SNCA*) or glucocerebrosidase (GBA) gene mutations.

The risk of dementia in patients with PARKIN mutations is reported to be very low (33). Nonetheless, neuropsychiatric features are reported, including anxiety, psychosis, panic attacks, depression, disturbed sexual behavioral, and obsessive–compulsive disorders. A recent cross-sectional study, comparing outcome in 21 PARKIN mutation carriers versus 23 idiopathic PD (IPD) patients, showed that PARKIN carriers had better performance, not only on the motor score but also on attention, memory, and visuospatial cognitive domains compared with non-carriers.

Leucine-rich repeat kinase 2 is a common genetic cause of PD and Gly2019Ser is the most frequent mutation. It was shown that Gly2019Ser carriers have lower PPD risk compared to IPD patients, particularly in the first 2 years of disease (34).

*SNCA* gene point mutations (A53T, A30P, and E46K), named PARK1, as well as duplications and triplications (PARK4), cause autosomal dominant PD with variable penetrance. Clinically, carriers may have rapid motor progression and frequent dementia, in particular, triplications result in earlier onset of PD and dementia (35). Moreover, functional *SNCA* SNPs are associated with different rates of motor progression in IPD (36).

Heterozygous GBA mutations are over-represented in cases of both familial and sporadic PD. Compared with IPD, patients carrying GBA mutations have been reported to have an earlier age at onset, more symmetrical clinical signs, and an increased incidence of neuropsychiatric disturbances (37). In 2012, Winder-Rhodes et al. demonstrated that in PD patients with single GBA mutations the risk of progression to dementia is more than five times higher that the one of GBA-negative patients, but the mechanism underlying this association has yet to be elucidated (38).

With the progress in genotyping and sequencing technologies, discovery of novel genetic markers associated with disease onset, progression, and response to treatment is expected. Nonetheless, the use of genetic markers to predict disease progression and specifically cognitive impairment in PD patients requires a great effort in collecting accurate and reproducible clinical data. To standardize procedures for acquisition, processing, and storage of clinical data which are often not homogeneous, is mandatory to improve the quality of the research. It is conceivable to collect more accurate indicators of dementia, through the systematic use of specific rating scales (e.g., DRS) that also take into account the interference of cognitive decline with the activities of daily living. This would eventually help in early identifying a subgroup of PD patients at risk of dementia to be enrolled in clinical trials of novel and more specific treatments. Finally, large collaborative studies involving several institutions will be required to prospectively validate the utility of these markers for clinical decision making.

## Conflict of Interest Statement

The authors declare that the research was conducted in the absence of any commercial or financial relationships that could be construed as a potential conflict of interest.

## References

[1] Hely MA Reid WG Adena MA Halliday GM Morris JG. The Sydney multicenter study of Parkinson’s disease: the inevitability of dementia at 20 years. Mov Disord (2008) 23:837–44. 10.1002/mds.21956 18307261

[2] Hughes AJ Daniel SE Kilford L Lees AJ. Accuracy of clinical diagnosis of idiopathic Parkinson’s disease: a clinico-pathological study of 100 cases. J Neurol Neurosurg Psychiatry (1992) 55:181–4. 10.1136/jnnp.55.3.181 1564476 PMC1014720

[3] Emre M Aarsland D Brown R Burn DJ Duyckaerts C Mizuno Y Clinical diagnostic criteria for dementia associated with Parkinson’s disease. Mov Disord (2007) 22:1689–707. 10.1002/mds.21507 17542011

[4] Rosenberg-Katz K Herman T Jacob Y Giladi N Hendler T Hausdorff JM. Gray matter atrophy distinguishes between Parkinson disease motor subtypes. Neurology (2013) 80:1476–84. 10.1212/WNL.0b013e31828cfaa4 23516323 PMC3662357

[5] Song SK Lee JE Park HJ Sohn YH Lee JD Lee PH. The pattern of cortical atrophy in patients with Parkinson’s disease according to cognitive status. Mov Disord (2011) 26:289–96. 10.1002/mds.23477 21370255

[6] Yong SW Yoon JK An YS Lee PH. A comparison of cerebral glucose metabolism in Parkinson’s disease, Parkinson’s disease dementia and dementia with Lewy bodies. Eur J Neurol (2007) 14:1357–62. 10.1111/j.1468-1331.2007.01977.x 17941855

[7] Arnaldi D Morbelli S Morrone E Campus C Nobili F. Cognitive impairment in degenerative parkinsonisms: role of radionuclide brain imaging. Q J Nucl Med Mol Imaging (2012) 56: 55–67. 22460160

[8] Cappellano G Carecchio M Fleetwood T Magistrelli L Cantello R Dianzani U Immunity and inflammation in neurodegenerative diseases. Am J Neurodegener Dis (2013) 2:89–107 23844334 PMC3703122

[9] Skillbäck T Zetterberg H Blennow K Mattsson N. Cerebrospinal fluid biomarkers for Alzheimer disease and subcortical axonal damage in 5,542 clinical samples. Alzheimers Res Ther (2013) 5:47. 10.1186/alzrt212 24479774 PMC3978733

[10] Carecchio M Fenoglio C Cortini F Comi C Benussi L Ghidoni R Cerebrospinal fluid biomarkers in progranulin mutations carriers. J Alzheimers Dis (2011) 27:781–90. 10.3233/JAD-2011-111046 21891865

[11] Sharma S Moon CS Khogali A Haidous A Chabenne A Ojo C Biomarkers in Parkinson’s disease (recent update). Neurochem Int (2013) 63:201–29. 10.1016/j.neuint.2013.06.005 23791710

[12] Parnetti L Tiraboschi P Lanari A Peducci M Padiglioni C D’Amore C Cerebrospinal fluid biomarkers in Parkinson’s disease with dementia and dementia with Lewy bodies. Biol Psychiatry (2008) 64(10):850–5. 10.1016/j.biopsych.2008.02.016 18395699

[13] Shi M Bradner J Hancock AM Chung KA Quinn JF Peskind ER Cerebrospinal fluid biomarkers for Parkinson disease diagnosis and progression. Ann Neurol (2011) 69:570–80. 10.1002/ana.22311 21400565 PMC3117674

[14] Siderowf A Xie SX Hurtig H Weintraub D Duda J Chen-Plotkin A CSF amyloid {beta} 1-42 predicts cognitive decline in Parkinson disease. Neurology (2010) 75:1055–61. 10.1212/WNL.0b013e3181f39a78 20720189 PMC2942062

[15] Comi C Carecchio M Chiocchetti A Nicola S Galimberti D Fenoglio C Osteopontin is increased in the cerebrospinal fluid of patients with Alzheimer’s disease and its levels correlate with cognitive decline. J Alzheimers Dis (2010) 19:1143–8. 10.3233/JAD-2010-1309 20308780

[16] Carecchio M Comi C. The role of osteopontin in neurodegenerative diseases. J Alzheimers Dis (2011) 25:179–85. 10.3233/JAD-2011-102151 21358042

[17] Maetzler W Berg D Schalamberidze N Melms A Schott K Mueller JC Osteopontin is elevated in Parkinson’s disease and its absence leads to reduced neurodegeneration in the MPTP model. Neurobiol Dis (2007) 25:473–82. 10.1016/j.nbd.2006.10.020 17188882

[18] Maetzler W Michelis J Tomiuk J Melms A Becker C Gasser T A single-nucleotide polymorphism of the osteopontin gene may contribute to a susceptibility to Lewy body disease. J Neural Transm (2009) 116:599–605. 10.1007/s00702-009-0209-x 19340392

[19] Chen-Plotkin AS Hu WT Siderowf A Weintraub D Goldmann Gross R Hurtig HI Plasma epidermal growth factor levels predict cognitive decline in Parkinson disease. Ann Neurol (2011) 69:655–63. 10.1002/ana.22271 21520231 PMC3155276

[20] González-Aramburu I Sánchez-Juan P Sierra M Fernández-Juan E Sánchez-Quintana C Berciano J Serum uric acid and risk of dementia in Parkinson’s disease. Parkinsonism Relat Disord (2014) 20:637–9. 10.1016/j.parkreldis.2014.02.023 24637121

[21] Song IU Kim JS Park IS Kim YD Cho HJ Chung SW Clinical significance of homocysteine (hcy) on dementia in Parkinson’s disease (PD). Arch Gerontol Geriatr (2013) 57:288–91. 10.1016/j.archger.2013.04.015 23706974

[22] Alberio T Pippione AC Comi C Olgiati S Cecconi D Zibetti M Dopaminergic therapies modulate the T-CELL proteome of patients with Parkinson’s disease. IUBMB Life (2012) 64:846–52. 10.1002/iub.1073 22815142

[23] Alberio T Pippione AC Zibetti M Olgiati S Cecconi D Comi C Discovery and verification of panels of T-lymphocyte proteins as biomarkers of Parkinson’s disease. Sci Rep (2012) 2:953. 10.1038/srep00953 23233872 PMC3518817

[24] Morley JF Xie SX Hurtig HI Stern MB Colcher A Horn S Genetic influences on cognitive decline in Parkinson’s disease. Mov Disord (2012) 27:512–8. 10.1002/mds.24946 22344634 PMC3323737

[25] Gatt AP Jones EL Francis PT Ballard C Bateman JM. Association of a polymorphism in mitochondrial transcription factor A (TFAM) with Parkinson’s disease dementia but not dementia with Lewy bodies. Neurosci Lett (2013) 557:177–80. 10.1016/j.neulet.2013.10.045 24184878

[26] Matsushita S Arai H Matsui T Yuzuriha T Urakami K Masaki T Brain-derived neurotrophic factor gene polymorphisms and Alzheimer’s disease. J Neural Transm (2005) 112:703–11. 10.1007/s00702-004-0210-3 15375678

[27] Guerini FR Beghi E Riboldazzi G Zangaglia R Pianezzola C Bono G BDNF Val66Met polymorphism is associated with cognitive impairment in Italian patients with Parkinson’s disease. Eur J Neurol (2009) 16:1240–5. 10.1111/j.1468-1331.2009.02706.x 19538209

[28] Huang X Chen P Kaufer DI Tröster AI Poole C. Apolipoprotein E and dementia in Parkinson disease: a meta-analysis. Arch Neurol (2006) 63:189–93. 10.1001/archneur.63.2.189 16476806

[29] Mata IF Leverenz JB Weintraub D Trojanowski JQ Hurtig HI Van Deerlin VM APOE, MAPT, and SNCA genes and cognitive performance in Parkinson disease. JAMA Neurol (2014). 10.1001/jamaneurol.2014.1455 25178429 PMC4227942

[30] Nombela C Rowe JB Winder-Rhodes SE. Genetic impact on cognition and brain function in newly diagnosed Parkinson’s disease: ICICLE-PD study. Brain (2014) 137(10):2743–58. 10.1093/brain/awu201 25080285 PMC4163033

[31] Setó-Salvia N Clarimón J Pagonabarraga J Pascual-Sedano B Campolongo A Combarros O Dementia risk in Parkinson disease: disentangling the role of MAPT haplotypes. Arch Neurol (2011) 68:359–64. 10.1001/archneurol.2011.17 21403021

[32] Ezquerra M Campdelacreu J Gaig C Compta Y Muñoz E Martí MJ Lack of association of APOE and tau polymorphisms with dementia in Parkinson’s disease. Neurosci Lett (2008) 448:20–3. 10.1016/j.neulet.2008.10.018 18930114

[33] Alcalay RN Caccappolo E Mejia-Santana H. Cognitive and motor function in long-duration PARKIN-associated Parkinson disease. JAMA Neurol (2014) 71(1):62–7. 10.1001/jamaneurol.2013.4498 24190026 PMC3947132

[34] Healy DG Falchi M O’Sullivan SS. Phenotype, genotype, and worldwide genetic penetrance of LRRK2-associated Parkinson’s disease: a case-control study. Lancet Neurol (2008) 7:583–90. 10.1016/S1474-4422(08)70117-0 18539534 PMC2832754

[35] Halliday GM Leverenz JB Schneider JS Adler CH. The neurobiological basis of cognitive impairment in Parkinson’s disease. Mov Disord (2014) 29:634–50. 10.1002/mds.25857 24757112 PMC4049032

[36] Ritz B Rhodes SL Bordelon Y Bronstein J. α-Synuclein genetic variants predict faster motor symptom progression in idiopathic Parkinson disease. PLoS One (2012) 7:e36199. 10.1371/journal.pone.0036199 22615757 PMC3352914

[37] Brockmann K Srulijes K Hauser AK Schulte C Csoti I Gasser T GBA-associated PD presents with nonmotor characteristics. Neurology (2011) 77:276–80. 10.1212/WNL.0b013e318225ab77 21734182

[38] Winder-Rhodes SE Evans JR Ban M Mason SL Williams-Gray CH Foltynie T Glucocerebrosidase mutations influence the natural history of Parkinson’s disease in a community-based incident cohort. Brain (2013) 136:392–9. 10.1093/brain/aws318 23413260

